# Long-Term Functional and Structural Renoprotection After Experimental Acute Kidney Injury in Subclinical Chronic Kidney Disease In Vivo

**DOI:** 10.3390/ijms26104616

**Published:** 2025-05-12

**Authors:** Sanjeeva Herath, Amy Y. M. Au, Kylie M. Taylor, Natasha Kapoor-Kaushik, Zoltán H. Endre, Jonathan H. Erlich

**Affiliations:** 1 School of Clinical Medicine, Faculty of Medicine and Health, University of New South Wales, Sydney, NSW 2052, Australia; sanjeeva.herath@unsw.edu.au (S.H.); amy.au@unsw.edu.au (A.Y.M.A.); j.erlich@unsw.edu.au (J.H.E.); 2 Department of Nephrology, Prince of Wales Hospital, Sydney, NSW 2031, Australia; 3 Electron Microscopy Unit, Mark Wainwright Analytical Centre, University of New South Wales, Sydney, NSW 2052, Australia

**Keywords:** acute kidney disease, chronic kidney disease, renoprotection, ischaemia–reperfusion injury, nicotinamide adenine dinucleotide, mitochondrial protection, kidney functional reserve

## Abstract

Subclinical chronic kidney disease (sCKD) predisposes one to acute kidney injury (AKI) and chronic kidney disease (CKD). Reduced kidney functional reserve (KFR) detects sCKD in preclinical studies and predicts AKI after cardiac surgery. We evaluated renal protection in a rat model of kidney injury where ischaemia–reperfusion injury (IRI) was induced after sCKD. Dual treatment boosting nicotinamide adenine dinucleotide (NAD) by nicotinamide riboside (NR) combined with the mitochondria-targeted antioxidant SkQR1 protected the KFR and reduced structural kidney damage, including markers of vascular integrity and the relative blood volume (rBV). The dual treatment upregulated *Sirt1* and *Nrf2,* increased the nuclear localisation of the mitochondrial biogenesis regulator PGC-1α and the mitochondrial protein marker COX4, and upregulated the antioxidant gene *NOQ1*. These observations suggest mitochondrial protection and modulation of the cellular redox state provided long-term structural and functional protection against kidney injury superimposed on background sCKD.

## 1. Introduction

Ischaemia–reperfusion injury (IRI) is a major cause of acute kidney injury (AKI) [1], with high morbidity and mortality [2,3]. The development of chronic kidney disease (CKD) after IRI-AKI depends on the duration of ischaemia [4] and premorbid kidney function [2,5]. As plasma creatinine does not increase beyond the normal range until the glomerular filtration rate (GFR) is reduced by more than 50%, it is an insensitive marker of significant kidney damage. Thus, CKD may not become clinically overt until more than 50% of the kidney parenchyma has been replaced by fibrosis [6,7]. When the plasma creatinine appears normal but there is extensive structural kidney injury, it is defined as subclinical CKD (sCKD), and this appears to precede most overt CKD [8,9,10]. After acute injury and loss of function, AKI recovery is limited by underlying sCKD, conferring a greater risk of progression to overt CKD [2,11]. As an increasingly older demographic presents for cardiac and other major surgeries, sCKD may explain the high incidence of post-surgical AKI, despite seemingly normal preoperative kidney function [12,13,14]. The detection of sCKD requires evidence of structural kidney injury, such as in a kidney biopsy or a kidney stress test, such as a kidney functional reserve (KFR) test.

KFR is defined as the increase in the GFR after a stimulus, most commonly an oral protein load [15]. Reduced KFR detects sCKD in an animal model [10]. KFR also has prognostic significance. Preoperative KFR detected by creatinine clearance predicted post-cardiac-surgery AKI with a 12-fold increased risk of AKI when the KFR was less than 15 mL/min (95% CI: 4.6–29.9; *p* < 0.001) [16].

Successful clinical studies demonstrating renal protection against AKI are limited to the recent demonstration of protection by perioperative amino acid infusion [17,18,19], which may recruit the KFR, enhancing renal perfusion to the cortex and medulla [20,21,22]. Mitochondrial protection by L-glutamine through decreased oxidative stress may also contribute to protection by amino acid infusion [23,24,25]. Hence, therapeutic strategies that target mitochondria for protection and enhance antioxidant pathways might similarly protect the KFR.

Models of IRI have been extensively used to evaluate kidney protection and characteristically demonstrate mitochondrial and oxidative injury [26,27,28,29]. However, IRI is usually applied in otherwise healthy animals, unlike the clinical cohort at the highest risk of AKI, namely, older adults with significant comorbidity [30,31,32]. Outcomes after IRI are also usually assessed close to the time of injury and may not reflect ongoing stimuli to injury and fibrosis.

In this study, we used a rat model that incorporated sCKD to simulate a common clinical condition in humans, particularly in elderly patients undergoing surgery with a risk of IRI. We hypothesised that mitochondrial protection by boosting nicotinamide adenine dinucleotide (NAD) using nicotinamide riboside (NR) supplementation combined with the mitochondria-targeted antioxidant SkQ group, including 10-(6′-plastoquinonyl) decylrhodamine 19 (SkQR1), would protect kidney structure and function, not just immediately but also 6 weeks after ischaemia when IRI was induced in the presence of underlying sCKD. We evaluated kidney protection by studies of function, including the KFR, and studies of structural integrity, including markers of vascular integrity and the relative blood volume (rBV).

## 2. Results

### 2.1. NR + SkQR1 Protected Stimulated GFR and KFR After IRI

Baseline and day-56 serum creatinine (sCr), creatinine clearance (CrCl), GFR, and urine biomarker data and sham control/vehicle control (D57 and 63) data were previously published as part of a larger cohort (n = 30 of 51) in Taylor et al.’s study [10], which confirmed the detection of sCKD by the KFR. As expected, IRI increased the mean sCr concentration and decreased CrCl 24 h after surgery (day 57) (Figure S1A,B).

**Figure 1:**
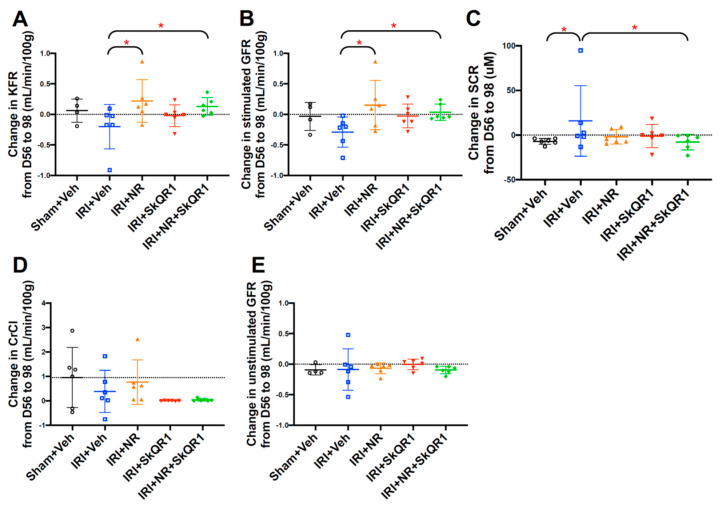
Changes in (**A**) kidney functional reserve (KFR), (**B**) stimulated glomerular filtration rate (GFR), (**C**) serum creatinine (sCr), (**D**) creatinine clearance (CrCl), and (**E**) unstimulated GFR from day 56 (subclinical chronic kidney disease (SCKD)) to day 98 in sham + vehicle (Veh), ischaemia–reperfusion injury (IRI) + Veh, IRI + nicotinamide riboside (NR), IRI + SkQR1, and IRI + NR + SkQR1 animals (n = 4–6 for each group). Means and standard deviations are shown. * *p* < 0.05.

An evaluation at 6 weeks after IRI (98 days after commencing the induction of sCKD) showed that the unstimulated (baseline) GFR was unchanged, but both the dual (NR + SkQR1) and single NR treatments protected the stimulated GFR and, thus, the KFR (Figure 1A–C and Figure S2). There was no significant change in sCr, the unstimulated GFR, or CrCl after IRI by day 98 (Figure 1D,E and Figure S2).

### 2.2. NR + SkQR1 Protected Relative Blood Volume (rBV) After IRI

**Figure 2:**
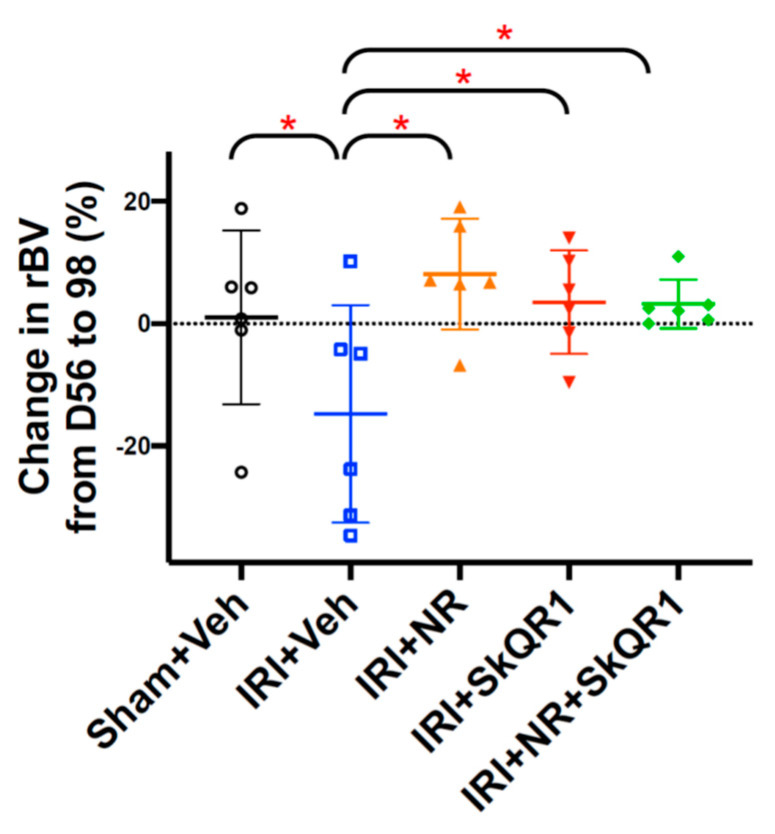
Nicotinamide riboside and SkQR1 protect relative blood volume (rBV) after IRI. Change in relative blood volume (rBV) from day 56 [subclinical chronic kidney disease (SCKD)] to day 98 in sham + vehicle (Veh), ischaemia–reperfusion injury (IRI) + Veh, IRI + nicotinamide riboside (NR), IRI + SkQR1, and IRI + NR + SkQR1 animals (n = 6 for each group). Means and standard deviations are shown. * *p* < 0.05.

The rBV was assessed from ultrasound flow measurements as a surrogate for the volumetric measurement of the vasculature since the loss of peritubular capillaries after IRI leads to the rarefaction of higher-order kidney vessels [33]. The rBV decreased by day 98 after IRI compared with the sham controls (Figure 2 and Figure S2D). Both the dual treatment (NR + SkQR1) and single (NR or SkQR1) treatments protected the rBV after IRI compared with the vehicle controls.

### 2.3. Kidney Injury After IRI Confirmed by Urine Biomarkers

**Figure 3:**
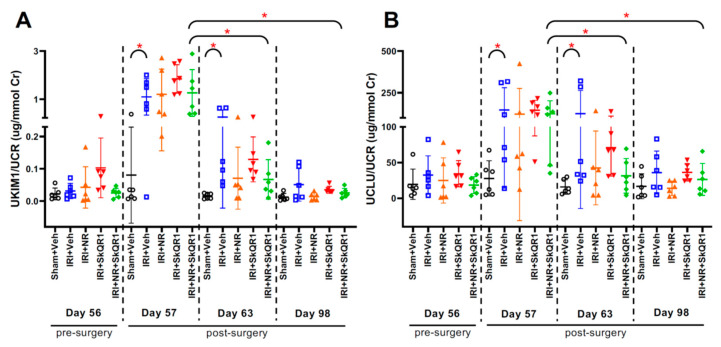
Urine biomarker changes after IRI. (**A**) Urinary kidney injury molecule-1 (uKIM-1)/urinary creatinine (uCr) and (**B**) urinary clusterin (uCLU)/uCr on days 56, 57, 63, and 98 in sham + vehicle (Veh), ischaemia–reperfusion injury (IRI) + Veh, IRI + nicotinamide riboside (NR), IRI + SkQR1, and IRI + NR + SkQR1 animals (n = 6 for each group). Means and standard deviations are shown. * *p* < 0.05.

Consistent with our previous study [10], the mean urine kidney injury molecule-1 (uKIM-1)/urine creatinine (uCr) and urine clusterin (uCLU)/uCr concentrations were increased 1 day after IRI in all groups. By day 98, the mean uKIM-1/uCr and uCLU/uCr remained significantly increased in the vehicle control animals compared with the sham animals, whereas the injury biomarkers rapidly returned to pre-IRI levels and were similar to the shams in the dual treatment (NR + SkQR1) group (Figure 3A,B).

### 2.4. NR + SkQR1 Treatment Protected Against Structural Damage

**Figure 4:**
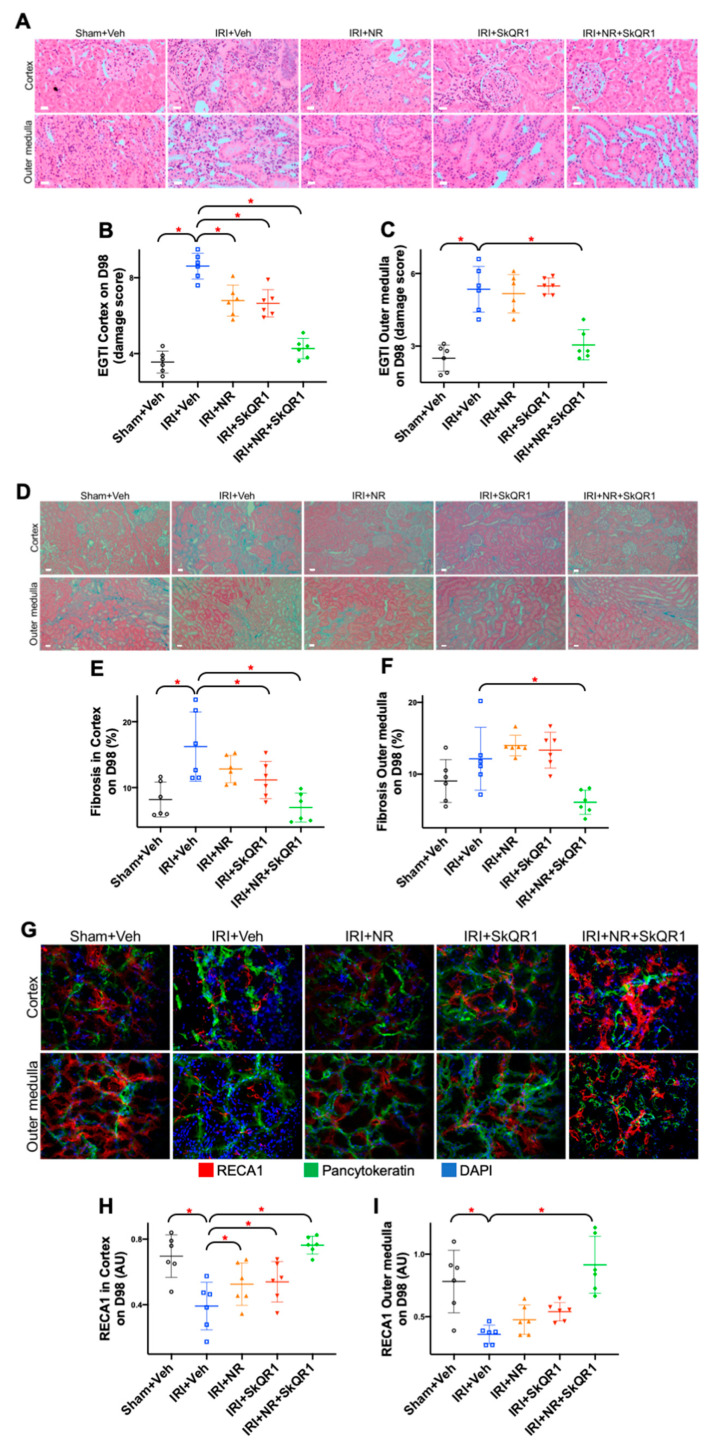
Nicotinamide riboside and SkQR1 protect against kidney structural damage after IRI. Representative images of (**A**) haematoxylin and eosin staining for (**B**,**C**) quantitation of endothelial, glomerular, tubular, and tubulo/interstitial (EGTI) damage score, (**D**) Masson’s trichrome stain for (**E**,**F**) quantitation of fibrosis and (**G**–**I**) peritubular endothelial cells (RECA1, red) in kidney cortex and outer medulla on day 98 in sham + vehicle (Veh), ischaemia–reperfusion injury (IRI) + Veh, IRI + nicotinamide riboside (NR), IRI + SkQR1, and IRI + NR + SkQR1 animals (n = 6 for each group). Pancytokeratin (green) and nucleus (blue) for RECA1 stain (red, for **C**). White bar is 10 microns (for **A**,**D**) and images taken at 400× magnification (for **G**). Means and standard deviations are shown. * *p* < 0.05.

Long-term (day 98) kidney damage was observed in the cortex and outer medulla as tubular necrosis and dilatation, interstitial inflammation, haemorrhage, and segmental glomerulosclerosis, 6 weeks after IRI and summarised as the endothelial, glomerular, tubular, and tubulo/interstitial (EGTI) score (Figure 4A–F). The single treatments reduced cortical injury, which was further reduced by the dual treatment. The dual treatment also reduced outer medullary fibrosis.

Rat endothelial cell antigen 1 (RECA1), normalised to pancytokeratin (to account for the tissue content), was used to identify the endothelium and, thus, as a marker of the peritubular capillary (PTC) density in the cortex (excluding the glomeruli) and in the outer medulla. RECA1 staining was reduced in the cortex and outer medulla after IRI, and this was prevented by the dual treatment (Figure 4G–I).

### 2.5. Mitochondrial Density Was Protected by NR + SkQR1

**Figure 5:**
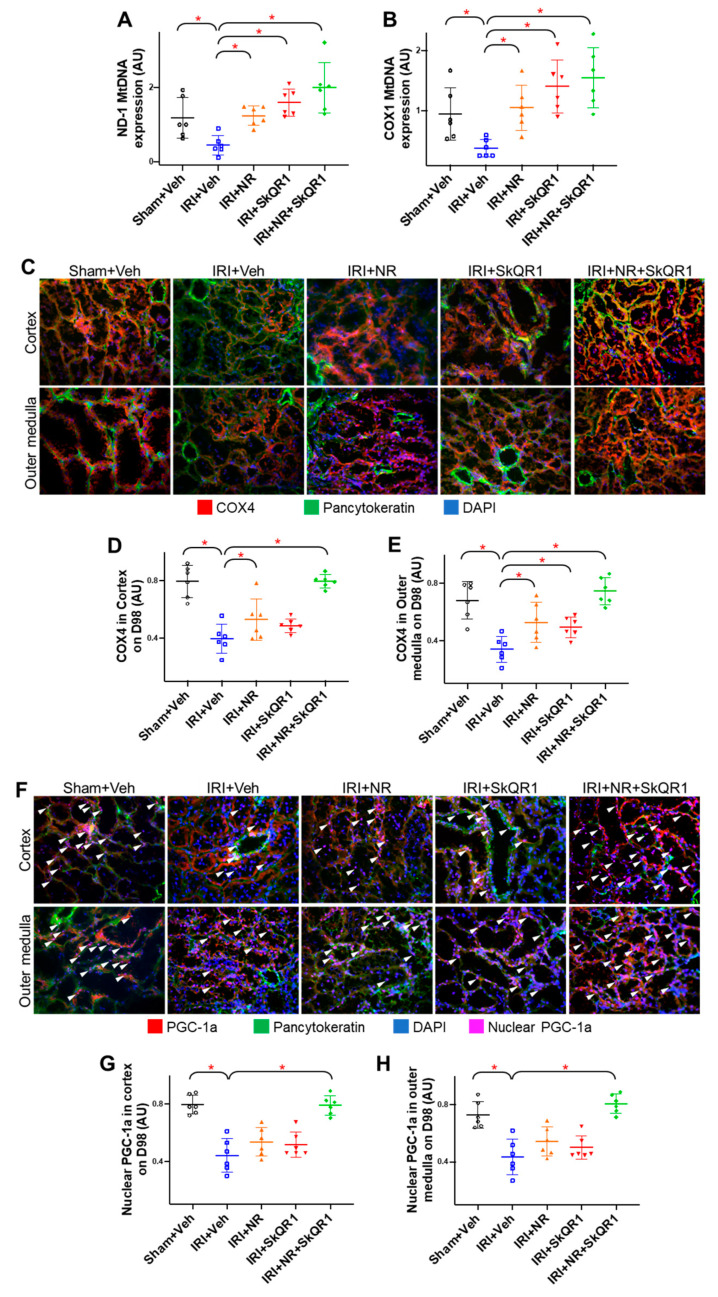
Kidney mitochondria-associated changes after IRI. (**A**) Mitochondrial NADH dehydrogenase 1 (Mt-ND-1) and (**B**) cytochrome c oxidase (Mt-COX-1) DNA kidney expression on day 98. Representative images and quantitation of (**C**–**E**) cytochrome c oxidase 4 (COX4, red) and (**F**–**H**) peroxisome proliferator-activated receptor-gamma coactivator-1α (PGC-1α, red) protein expression in kidney cortex and outer medulla on day 98 in sham + vehicle (Veh), ischaemia–reperfusion injury (IRI) + Veh, IRI + nicotinamide riboside (NR), IRI + SkQR1, and IRI + NR + SkQR1 animals (n = 6 for each group). Pancytokeratin (green) and nucleus (blue) (for **C**,**F**) and co-localisations for PGC-1α and nucleus (nuclear PGC-1a, pink) are as indicated (white arrows) (for **F**). Images taken at 400× magnification (**C**,**F**). Means and standard deviations are shown. * *p* < 0.05.

The mitochondrial density was quantified using the expression of mitochondrial DNA genes, NADH–ubiquinone oxidoreductase chain 1 (Mt-ND-1), and cytochrome c oxidase subunit 1 (Mt-COX1), and with the expression of the mitochondrial cytochrome c oxidase subunit 4 (COX4) protein. The mean Mt-ND-1 and Mt-COX1 DNA expression decreased after IRI (the vehicle compared with the sham controls) (Figure 5A,B). Both the single and dual treatments increased Mt-ND-1 and Mt-COX1 DNA expression. The COX4 protein expression followed a similar pattern and was decreased in both the cortex and outer medulla in the vehicle controls (Figure 5C–E) on day 98. Both the single and dual treatments protected Mt-COX4, with the greatest protection seen with the dual treatment.

The quantitation of peroxisome proliferator-activated receptor-gamma coactivator (PGC)-1α nuclear localisation was evaluated as a surrogate for PGC-1α activity [34]. The mean PGC-1α nuclear localisation was decreased in both the cortex and outer medulla on day 98 in the vehicle controls compared with the sham controls (Figure 5E–H). The dual treatment increased the mean PGC-1α nuclear localisation similarly to the sham control group.

### 2.6. Kidney Redox State and Treatment

**Figure 6:**
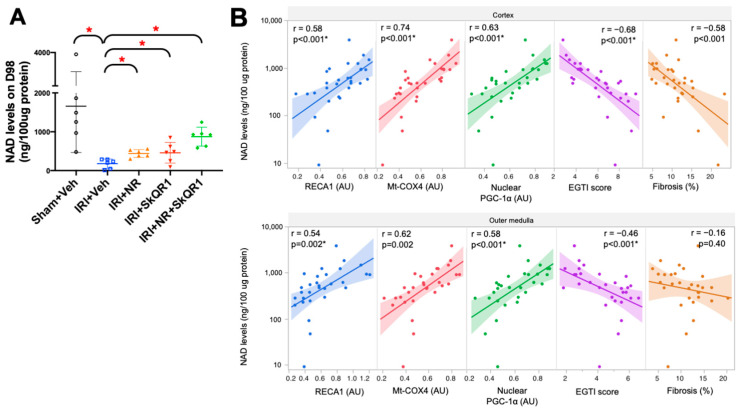
Nicotinamide adenine dinucleotide (NAD) levels on day 98. (**A**) Nicotinamide adenine dinucleotide (NAD) levels in sham + vehicle (Veh), ischaemia–reperfusion injury (IRI) + Veh, IRI + nicotinamide riboside (NR), IRI + SkQR1, and IRI + NR + SkQR1 animals on day 98 (n = 6 for each group). Means and standard deviations are shown. * *p* < 0.05. (**B**) Correlation of NAD levels with peritubular endothelial cell expression (RECA1), mitochondrial cytochrome c oxidase 4 expression (Mt-COX4), nuclear peroxisome proliferator-activated receptor-gamma coactivator-1α (PGC-1α) expression, EGTI score, and fibrosis in cortex (top panel) and outer medulla (bottom panel). * *p* < 0.05; r, Pearson’s correlation.

Nicotinamide adenine dinucleotide (NAD) levels were significantly decreased in the kidneys in the vehicle control group (Figure 6A). NR supplementation increased NAD levels in the single and dual treatments on day 98. NAD levels were correlated positively with RECA1, PGC-1α nuclear localisation, and Mt-COX4 expression and were correlated negatively with the tubular injury score, EGTI, and fibrosis (Figure 6B). These correlations suggest NAD depletion contributed to the loss of the endothelium and fibrosis associated with mitochondrial depletion in the untreated animals.

### 2.7. Correlation of Functional Markers with Structural Markers

**Figure 7:**
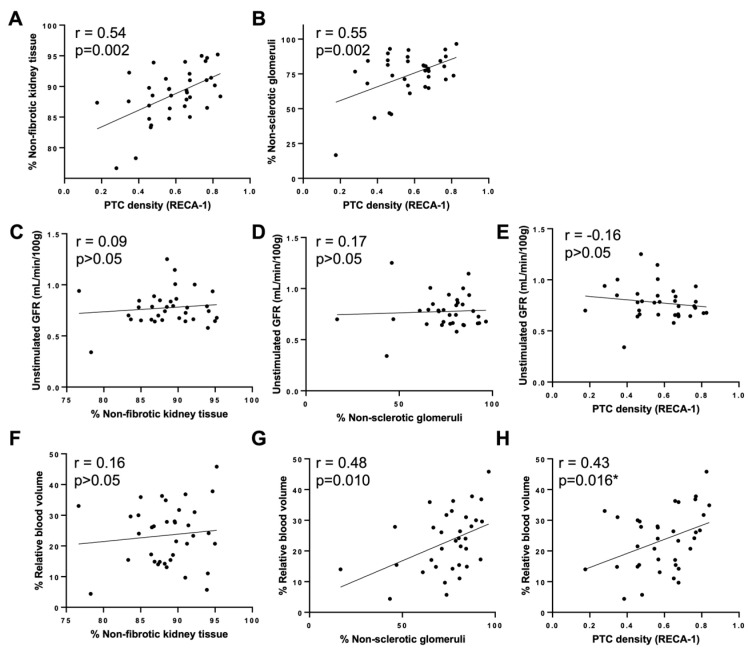
Correlation of peritubular capillary (PTC) density with (**A**) % non-fibrotic and (**B**) % non-sclerotic kidney tissue; unstimulated GFR with (**C**) % non-fibrotic kidney tissue, (**D**) % non-sclerotic kidney tissue, and (**E**) PTC density; and % relative blood volume with (**F**) % non-fibrotic kidney tissue, (**G**) % non-sclerotic kidney tissue, and (**H**) PTC density. * *p* < 0.05; r, Pearson’s correlation.

On day 98, the PTC density (RECA1 expression) was correlated with non-fibrotic tissue (r = 0.54; Figure 7A) and non-sclerotic glomeruli (r = 0.55; Figure 7B). There was no correlation between the unstimulated GFR and non-fibrotic tissue, non-sclerotic glomeruli, or PTC density (Figure 7C–E). The rBV was correlated with non-sclerotic glomeruli (r = 0.48; Figure 7G) and the PTC density (r = 0.43; Figure 7H) but not with non-fibrotic tissue (Figure 7F).

### 2.8. Identification of Protection Pathways Against IRI

**Figure 8:**
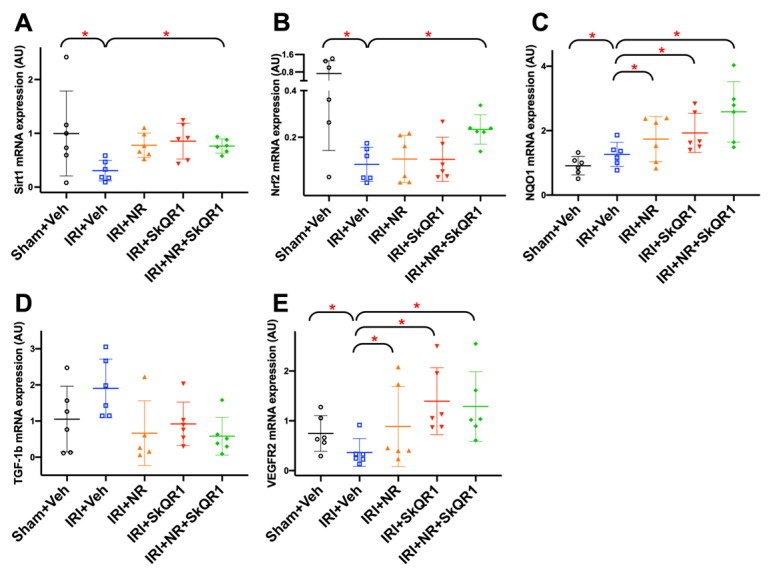
(**A**) Sirtuin 1 (Sirt1), (**B**) nuclear factor erythroid 2-related factor 2 (Nrf2), (**C**) NAD(P)H quinone dehydrogenase 1 (NQO1), (**D**) transforming growth factor 1 beta (TGF-1β), and (**E**) vascular endothelial growth factor receptor 2 (VEGFR2) kidney mRNA expression on day 98 in sham + vehicle (Veh), ischaemia–reperfusion injury (IRI) + Veh, IRI + nicotinamide riboside (NR), IRI + SkQR1, and IRI + NR + SkQR1 animals on day 98 (n = 6 for each group). Means and standard deviations are shown. * *p* < 0.05.

The expression of specific genes associated with PGC-1α activity and mitochondrial biogenesis, sirtuin (Sirt) 1 and nuclear factor erythroid 2-related factor (Nrf) 2, was decreased after IRI (Figure 8A,B), and there was an increase in NAD(P)H quinone oxidoreductase 1 (NQO1) (Figure 8C). The dual treatment (NR + SkQR1) increased Sirt1, Nrf2, and NQO1 expressions on day 98. Consistent with histological markers, transforming growth factor (TGF)-1β and vascular endothelial growth factor receptor (VEGFR)-2 increased with IRI, and the dual treatment decreased both on day 98 (Figure 8D,E).

The histological biomarkers (EGTI score, fibrosis, and Mt-COX1 DNA) assessed on day 98 discriminated the vehicle control from the dual-treatment group with AUCs of 1.0 in univariable analyses (Table 1). Multivariable analysis using non-invasive biomarkers on day 98, uKIM-1/uCr, uCLU/uCr, rBV, and KFR, identified the vehicle controls (n = 6) from all treated animals (n = 18), with an AUC of 1.0 (95% CI: 0.98–1.0) (Table 1).

## 3. Discussion

While previous studies have shown that targeting the kidney mitochondria provides early protection against kidney injury in AKI [27,35], this is the first study to show that prevention of mitochondrial injury provides long-term protection and does so even in the presence of background kidney damage, an experimental model that mimics the clinical reality of AKI in increasingly older adults with background comorbidity, including CKD. Preventative treatment with NR and SkQR1 provided functional, haemodynamic, and structural protection at 6 weeks post-IRI in animals with background sCKD. KFR and rBV were protected, while histological damage, rarefaction of the microvasculature, and mitochondrial dysfunction were ameliorated compared with the control animals. The protection was most pronounced with the dual NR + SkQR1 treatment.

Animal and clinical studies have shown that AKI and CKD are interconnected syndromes on a continuum of injury, with each being a risk factor for the other and manifested by histological damage, microvascular rarefaction, and a reduced GFR [2,33]. Subjects with underlying kidney impairment, therefore, have a greater risk of progression to overt CKD if exposed to an AKI event [1,4]. Where there is a high risk of IRI-induced AKI and the timing of exposure is known, such as before major surgery, the opportunity to ameliorate IRI-induced AKI may prevent CKD progression.

Mitochondrial protection using NR and SkQR1 has been shown to ameliorate AKI induced by IRI, nephrotoxicity, or sepsis [36,37,38,39]. SkQR1 accumulates in mitochondria in cell culture and protects mitochondrial integrity by stabilising the voltage gradient [40]. NAD+ is a cofactor for multiple enzymes involved in energy metabolism [41], cell antioxidant responses [42], and cell survival [43]. This study investigated NR and SkQR1 in the prevention of IRI-induced AKI on a background of sCKD [8,10]. Dual treatment with NR and SQR1 improved NAD levels, highlighting improved cellular energetics as one explanation for its efficacy.

The single and dual treatments provided long-term (6 weeks) functional (GFR and KFR) and structural kidney protection, including the preservation of the microvasculature and mitochondrial density, protecting against kidney injury almost to the degree found in sham-operated controls. Our findings support prior studies showing that mitochondrial stabilisers provide nephroprotection [34,44,45,46] and highlight the utility of strategies to protect the kidneys against further injury when AKI events are likely. Consistent with this, mitochondria-targeted ubiquinol MitoQ has been safely administered in humans in phase II trials, and a study in healthy adults showed no evidence of nephrotoxicity [47,48,49]. MitoQ administration during cold storage prior to IRI-induced AKI prevented kidney damage [44,50,51,52] and may optimise transplant protection if administered at kidney retrieval [53].

The dual NR + SkQR1 treatment improved the stimulated GFR and KFR 6 weeks after IRI was paralleled by the preservation of the rBV, consistent with the improved vascular function, such as flow-mediated dilation and microvascular function, in CKD patients treated with MitoQ to reduce mitochondrial reactive oxygen species (ROS) [54]. SkQR1 also improves renal blood flow and reduces renal vascular resistance after reperfusion [37]. The mechanisms underlying the KFR are complex and likely involve the modulation of tubuloglomerular feedback, afferent arteriolar vasodilatation, nitric oxide, glucagon, and vasodilator prostaglandins [55]. In our studies, the PTC density was correlated with the non-sclerotic glomerular number and non-fibrotic parenchyma, and similarly, the rBV was correlated with non-sclerotic glomeruli and the PTC density. Glomerular and renal interstitial fibrosis and the reduction in the rBV are consistent with the microvascular rarefaction characteristic of progressive kidney disease [1,33]. Although there was no direct correlation between ultrasound-measured renal perfusion and the KFR in this study, the protection of endothelial cells and the PTC density, with the subsequent protection of renal perfusion, could be a mechanism by which mitochondrial protection preserved the KFR and stimulated GFR.

Sirt1-PGC1α-mitochondrial biogenesis and Nrf2-antioxidant response pathways also contributed to nephroprotection in these studies. NR treatment was intended to increase NAD-dependent enzymes, such as Sirt1, and, in turn, increase PGC1α and Nrf2 activity to protect against IRI-induced reduced mitochondrial biogenesis. PGC-1α activity was protected with an increase in nuclear PGC-1α with the single treatment compared with the controls, while the dual treatment was most protective. Mt-COX4 protein expression and mitochondrial DNA (Mt-ND-1 and Mt-COX-1 DNA expression) were similarly protected by the single and dual treatments.

All biomarkers of injury were significantly raised on day 57 (post-IRI day 1), confirming the establishment of adequate IRI by clamping. Urinary KIM1 and clusterin were elevated in the vehicle control group but reached pre-IRI levels in the dual-treatment group, which indicates ongoing injury in the vehicle group compared with the dual-treatment group [8]. Although injury biomarkers by themselves were not able to differentiate between the vehicle and treatment groups, when combined with an additional non-invasive measure, such as rBV or KFR, the model was able to identify the treated groups (Table 1). This could be utilised to predict the response to mitochondrial-targeted treatment when conducting human studies.

There were several limitations to this study, including its small sample size based on the published effect sizes [42] and significant inter-subject variation. The effect of IRI on normal animals and mitochondrial protection has already been shown to preserve normal kidney structure and function against ischaemic and other insults in normal kidneys [56]. The strengths of this study included using a model with background subclinical CKD reflecting the adult clinical population at high risk of AKI and a longitudinal design with repeated measures of kidney function and blood flow, and a mixed model analysis of data to consider the between- and within-group variability.

In conclusion, NR and SkQR1 protected against IRI-induced reductions in the stimulated GFR and KFR in a model with sCKD. This protection was demonstrated by a reduction in fibrosis, the preservation of the microvasculature, and a reduction in damage biomarkers. The protection appeared to be mediated by the protection of mitochondrial biogenesis, reflected by the preservation of mitochondrial DNA, proteins, and transcriptional regulators. Validation of renoprotection by targeting mitochondrial biogenesis awaits further studies, including studies involving other aetiologies of AKI.

## 4. Materials and Methods

### 4.1. Animals

Animal studies were conducted in accordance with the Australian Code for the Care and Use of Animals for Scientific Purposes (National Health and Medical Research Council, 2013) and approved by the Animal Ethics Committee at the University of New South Wales (20/9A, 19/109A).

Seven-week-old male Sprague Dawley (SD) rats (Animal Resource Centre, Perth, Western Australia) weighing approximately 250–300 g were used for all studies. The rats were acclimatised for 1 week prior to the commencement of the experiments. The rats had ad libitum access to water and commercial rodent chow (Gordon’s chow, Specialty Feeds, Australia); this provided 14.2 kJ/g of energy and 59.9% of nutrition as carbohydrates, 19% as protein, and 4.6% as total fat (normal chow).

### 4.2. sCKD Induction and IRI Protocol

sCKD was induced by feeding 0.25% adenine (Sigma-Aldrich, Melbourne, Australia) in normal chow for 28 days, followed by normal chow without adenine for a further 28 days (n = 30) (Figure S3) [8,10]. As in previous studies, only male rats were used due to the gender differences in renal outcomes of adenine-fed animals, according to Diwan, V. et al. [57]. The sCKD rats were randomly distributed among the experimental groups and then underwent bilateral IRI or sham surgery (sham, n = 6; IRI, n = 24) on day 56. The renal pedicles were clamped for 30 min to induce IRI, as previously described [10]. Sham-operated animals underwent the same surgery without vascular clamping. On day 98, the animals were anaesthetised with isoflurane/O2 and then exsanguinated at renal removal, thus euthanising the animals, as previously described [10].

### 4.3. Treatment Groups

Nicotinamide riboside (NR) (0.1% *w*/*w*) was mixed with normal chow and provided ad libitum from day 53 to 98. As NR administration in the chow was simple, cheap, and non-invasive, this was commenced 3 days prior to IRI, mirroring a potential clinical approach. SkQR1 (100 nmol/kg in normal saline) or an equal volume of the SkQR1 vehicle was administered intraperitoneally (IP) 3 h prior to the sham or IRI surgery on day 56 and then 1 h, 24, 48, and 72 h post-surgery. The sham-operated animals were administered the IP vehicle and fed normal chow (sham control: sham + Veh; n = 6). The IRI animals were either administered the vehicle and fed normal chow (vehicle control: IRI + Veh; n = 6), administered the vehicle and fed the NR diet (NR treatment: IRI + NR; n = 6), administered SkQR1 and fed normal chow (SkQR1 treatment: IRI + SkQR1), or administered SkQR1 and fed the NR diet (dual treatment: IRI + NR + SkQR1; n = 6). The sample size was calculated using the G power software (version 3.1.9.2) [50] based on Cohen’s principles [51,52,53]. The power, the least-significant value, and the lowest number to be treated based on *p* (alpha) = 0.05 and the SD of 1.6 using the effect size of 2.5 units of difference in the fibrosis scores at 8 weeks after IRI observed in our previous (unpublished) study looking at adenine- and non-adenine-fed rats indicated that only 3 rats were needed per group (JMP-Pro). Six rats would provide a power of 0.99. The results from our control sCKD + IRI group were consistent with our previous study [3], so we continued with n = 6 animals for each treatment group.

### 4.4. GFR Measurements

The GFR was determined from the clearance of FITC–sinistrin (Medibeacon, Mannheim, Germany), as previously described [10,58]. Stimulated GFR measurements were obtained following protein gavage (5 g/kg of meat extract; Merck Millipore, Burlington, MA, USA), as previously described [10]. Stimulated and unstimulated GFR measurements were conducted on days 0–1 (baseline), 52–53 (labelled as day 56, prior to the NR diet), and days 96–97 (labelled as day 98). KFR was defined as [stimulated GFR − unstimulated GFR].

### 4.5. Biomarker Measurements

Blood and urine were collected prior to surgery (day 56), 1 day after surgery (day 57), 1 week after surgery (day 63), and 6 weeks after surgery (day 98). The samples were processed as described in the Supplementary Methods. The urinary biomarkers were measured with a Bio-Plex 200 system (Bio-Rad Laboratories, Hercules, CA, USA) with Bio-Plex Pro RBM Rat Kidney Toxicity Panel 1. sCr and urinary creatinine (uCr) were measured by the creatinine enzymatic assay (Thermo Fisher Scientific, Waltham, MA, USA) using a Konelab 20XT analyser (Thermo Fisher Scientific) or a microplate. The urinary biomarkers were normalised to uCr, as previously described [10].

### 4.6. Histopathology

Mid-coronal slices (5 mm thickness) were processed as previously described [8]. Image acquisition was performed by the Cell Sens Dimension^®^ software version 2.3 (Evident Scientific, Tokyo, Japan) at a 2560 × 1920 resolution and a 200× objective for haematoxylin and eosin (H&E)-stained sections and a 100× objective for Masson’s trichrome-stained sections. For each rat, 30 cortical and 30 medullary images were captured and analysed using Adobe Photoshop (version 23.2.2, Adobe Systems Corporation, San Jose, CA, USA). The tubular damage score per image [endothelial, glomerular, tubular, tubulo-interstitial (EGTI) score] was assessed from markers of tubulointerstitial damage (necrosis, tubular dilatation, cytoplasmic vacuolisation, and interstitial inflammation) [59]. Fibrosis was assessed as described by Dahab et al. [60], and the percentage of fibrosis was calculated as the area of fibrosis divided by the total area of kidney tissue.

### 4.7. Immunofluorescence

The kidney sections were embedded in optimal cutting temperature (OCT) embedding compound (Tissue-Tek, Sakura Finetek, Torrance, CA, USA), immediately frozen in liquid nitrogen, and then stored at −80 °C. Immunofluorescence measurements were performed on 6 μm cryosections mounted on positively charged slides. Pancytokeratin was used as a marker of the tubular epithelium to account for the tissue content in each section. Double staining was performed to investigate the co-occurrence of peroxisome proliferator-activated receptor gamma coactivator 1-alpha (PGC-1α)/pancytokeratin, mitochondrial marker cytochrome c oxidase subunit 4 isoform 1 (COX4)/pancytokeratin, and rat endothelial cell antibody (RECA1)/pancytokeratin with primary antibodies and the respective secondary antibodies with divergent wavelengths, as detailed in Table S1. The negative controls consisted of phosphate-buffered saline or irrelevant matched immunoglobulin G instead of primary antibodies and remained negative. Fluorescence microscopy was performed using an Olympus BX 51 fluorescent microscope (Teledyne Photometrics, Tucson, AZ, USA) with a Retiga LUMO CCD high-resolution camera feeding into Cell Sens Dimension^®^ software version 2.3 (Evident Scientific). All immunofluorescence was imaged with a ×400 objective. RECA1 was imaged by setting the exposure at 500 ms, PGC-1α and COX4 at 100 ms, and DAPI at 10 ms. The image acquisition was performed with binning at 2 × 2.

### 4.8. Quantitation of Protein of Interest

The positive area in each image (expressed in pixels) was quantified using the ImageJ software (version 1.53t, National Institute of Health, Bethesda, MD, USA). Due to the heterogeneity of illumination and lower signal-to-noise ratio, background subtraction was performed with minor modifications using dark and fluorescent images, as described by Model MA, 2006 [61]. All proteins of interest for each rat were expressed as fold changes compared with IRI, the vehicle group’s global average. Thirty micrographs (each micrograph is described as a ‘section’ below) were imaged in each cortex, medulla, and cortical section containing glomeruli.

Since the simple RECA1 area fraction underestimates the vascular volume, a previously published macro [62] was used to fill the peritubular capillary luminal volume once the visualised RECA1 was manually marked by thresholding for each section. Then, the pancytokeratin area fraction was obtained for the same section. The normalised RECA1-stained vascular area was calculated using the following equation: normalised RECA1 = macro-corrected RECA1-stained vascular area/pancytokeratin area fraction.

The fraction of PGC-1α co-localised to the nucleus was obtained by the ‘segmentation approach’, as described in [63], with minor modifications. Briefly, (i) masks were generated for pancytokeratin and total PGC-1α, separately, and the fractional areas were measured (Ex and total PGC-1α, respectively). (ii) Then, a composite of PGC-1α and the DAPI counterstain was made. This image was segmented, and the DAPI areas were subtracted (from the thresholding). The remaining mask consisted only of cytoplasmic PGC-1α. (iii) A DAPI count was obtained, which corresponded to the total cell count for that section (cell count). The normalised nuclear fraction of PGC-1α was then nuclear PGC-1α = [(total PGC-1α − cytoplasmic PGC-1α)/total PGC-1α] × [pancytokeratin (E)/ total DAPI count].

COX4 was co-stained with pancytokeratin using rabbit primary and mouse primary antibodies, respectively, and then visualised with the corresponding donkey anti-rabbit 488 and anti-mouse 594 secondaries, as detailed in Table S1. The normalised COX 4 fraction was also calculated using the method described above for nuclear PGC-1α.

### 4.9. NAD Measurement

The NAD measurement was performed using kidney extracts (six rats per group) using the NAD/NADH colourimetric assay kit (ab65348, Abcam, Cambridge, UK) as per the manufacturer’s instructions and read at 120 min.

### 4.10. Vascular Ultrasound

Relative quantitative information on kidney perfusion and haemodynamics was obtained using an ultra-high frequency linear array transducer, MX 400, at 20–46 MHz (with a centre transmit of 30 MHz and an axial resolution of 50 µm) coupled to a Vevo^®^ 3100 high-resolution imaging system (FUJIFILM, VisualSonics, Toronto, ON, Canada) in anaesthetised animals (with 4% isoflurane), supine on a heating pad at 37 °C (FUJIFILM). Vascular ultrasound measurements were performed on days 0 (baseline), 56 (the day of IRI), 57 (the day after IRI), and 98 (the study endpoint). Two-dimensional (2D) B-mode and three-dimensional (3D) power and colour Doppler measurements of the vasculature were obtained at a depth of 3–4 cm at a frame rate of 50 frames/sec using an ultrasound probe fixed to a 3D motor (FUJIFILM). All images were analysed by Vevo lab (FUJIFILM), and the total kidney volume was manually delineated for each frame. The vascular flow in each frame was also used to calculate the percentage of vascularity for the whole kidney. Power Doppler measures of vascular flow were used as a surrogate for the volumetric measurement of the vasculature. The relative blood volume (rBV) was then calculated as the percentage of total kidney volume occupied by the vascular volume.

### 4.11. RNA Extraction, cDNA Synthesis, and qPCRs

Total RNA was isolated from the homogenised rat tissue samples using the TRIsure kit, and cDNA was synthesised using the Tetro cDNA synthesis kit as per the manufacturer’s instructions (Meridian Bioscience, Cincinnati, OH, USA). The primers were designed by Beacon Designer (Premier Biosoft, San Francisco, CA, USA) to verify the specificity (BLAST analysis), the exclusion of genomic DNA, and the avoidance of structural folding areas and cross-homology. All primers (Table S2) were optimised to ensure specificity by melt curve analysis and an amplification efficiency of 90–110%. RT-qPCR was performed using a Sensifast SYBR No-Rox mastermix (Meridian Bioscience) as per the manufacturer’s instructions. RT-qPCR was performed in triplicate, and n= 6 animals per treatment group on the CFX Connect (Bio-Rad Laboratories) and averaged. Each gene was normalised to the geometric mean of β-actin (ACTB) and polyA-binding protein, nuclear 1 (PABPN1) house-keeping gene expression by the 2^∆∆CT^ value method. The geometric mean of ACTB × PABPN1 was deemed to be the optimum normalisation factor for rat IRI in sCKD studies in our previous study [64].

### 4.12. Mitochondrial DNA PCR

Genomic DNA was isolated with the Isolate II genomic DNA kit (Meridian Bioscience) using 25 mg of frozen kidney cortex and medulla. The DNA was quantified using the Quant-iT Picogreen dsDNA assay kit (Thermo Fisher Scientific). PCRs were performed in triplicate and averaged (n = 6 per group). The Mt-ND1 and Mt-COX1 DNA genes were then normalised to the geometric mean of the nuclear-encoded NADH dehydrogenase [ubiquinone] flavoprotein 1 (NDUFV1) DNA and the ubiquitin C (UBC) DNA gene using the 2^∆∆Ct^ method.

### 4.13. Statistics

Statistical analyses were performed in JMP Version 16 (SAS Institute, Cary, NC, USA); the data were transformed where appropriate. For all analyses, the primary actual values were used for model hypothesis testing. If the data residuals were normally distributed and the normal quintile plot fell within acceptable limits, the data were not transformed. Otherwise, the data were log-transformed, the log quintile plots were checked for normality, and parametric analysis was performed. However, all the data were presented as raw, or the non-transformed data were calculated, and the transformed data were only used in the modelling as described above. Statistical modelling was performed with mixed-model analysis for samples with repeated measures, standard ANOVA for group analysis with covariates, where applicable, and logistic regression with or without LASSO, which identified the penalty for a small n and more regressors (variables). The outcomes of the single treatment groups (NR, SkQR1, and NR + SkQR1; n = 6 per group) compared with the control group (n = 6) were the primary analysis. A *p*-value of < 0.05 from Tukey’s test was considered significant. Pearson’s correlation of r > 0.2 and a *p*-value of < 0.05 were considered significant. Nominal logistic models were performed to distinguish the groups.

## Figures and Tables

**Table 1:** Regression models using histological and mitochondrial biomarkers on day 98 to identify the dual-treated animals (NR + SkQR1) from the vehicle group and using non-invasive biomarkers to identify all treated animals (NR, SkQR1, and NR + SkQR1) from the vehicle group.

Dual Treatment (NR + SkQR1; n = 6) vs. Vehicle Group (n = 6)	Univariable Analysis	AUC (95% CI)
Histological or mitochondrial biomarkers (nominal logistic regression model)	EGTI score (cortex)	1.00 (1.00–1.00) *
	Fibrosis (cortex)	1.00 (1.00–1.00) *
	Mt-COX1 DNA	1.00 (1.00–1.00) *
**All Treated (NR, SkQR1, and NR + SkQR1; n = 18) vs** **.** **Vehicle Group (n = 6)**	**Multivariable Analysis**	**AUC (95% CI)**
Non-invasive biomarkers (LASSO regression model)	UKIM1/UCR, UCLU/UCR, KFR, and rBV	1.00 (0.98–1.00) *

Notes: AUC, area under the curve; BV, blood volume; CI, confidence interval; EGTI, endothelial, glomerular, tubular, and tubulo/interstitial; IRI, ischaemia–reperfusion injury; KFR, kidney functional reserve; LASSO, least absolute shrinkage and selection operator; Mt-COX1, mitochondrial cyclooxygenase 1; NR, nicotinamide riboside; UCLU, urinary clusterin; UCR, urinary creatinine; UKIM1, urinary kidney injury molecule-1; * *p* < 0.05.

## Data Availability

The additional data supporting this manuscript are available from the corresponding author upon request.

## Supplementary Materials

The following supporting information can be downloaded at https://www.mdpi.com/article/10.3390/ijms26104616/s1.
