# Supplementary material for: Long-Term Functional and Structural Renoprotection After Experimental Acute Kidney Injury in Subclinical Chronic Kidney Disease In Vivo
Source: Int J Mol Sci. 2025 May 12;26(10):4616. doi: 10.3390/ijms26104616 (PMC12111561; doi:10.3390/ijms26104616)
Supplement: Supplementary file 1 [file ijms-26-04616-s001.zip › ijms-3583228-supplementary.pdf]

SUPPLEMENTARY MATERIAL

Contents

Supplementary Table S1. Immunofluorescence reagents
Supplementary Table S2. qPCR primers
Supplementary Figure S1. sCr and CrCl changes after IRI on day 57, 63 and 98.
Supplementary Figure S2. KFR, stimulated GFR, unstimulated GFR and rBV on day 56 and 98.
Supplementary Figure S3. Details of experimental protocol.

Supplementary Table S1. Immunofluorescence reagents

Antibodies/Reagents	Company	Catalogue No.	Dilution
Mouse anti Rat endothelial cell antibody 1 (RECA1)	Abcam	ab9774	1:40
Rabbit anti pancytokeratin antibody	Abcam	ab9377	1:150
Mouse anti pancytokeratin antibody (C11)	Abcam	ab7753	1:150
Rabbit anti Cox 4 antibody	Abcam	ab209727	1:100
Rabbit PGC1α antibody	Novus Biologicals	NBP1-04676	1:100
Donkey anti mouse secondary IgG antibody Alexaflour™ 488	Thermofisher	A21202	1:250
Donkey anti mouse secondary IgG antibody Alexaflour™ 594	Thermofisher	A21203	1:250
Donkey anti rabbit secondary IgG antibody Alexaflour™ 488	Thermofisher	A21206	1:250
Donkey anti rabbit secondary IgG antibody Alexaflour™ 594	Thermofisher	A21207	1:200
DAPI	Thermofisher	1306	1:1000
Donkey serum	Abcam	ab7475	1:10-1:100
Prolong diamond™ antifade mountant Thermofisher	Thermofisher	P36965	
Superfrost-OT positively charged slides	Thermofisher	J1800AMNZ	

Supplementary Table S2. qPCR primers

Primer name	Forward primer sequence (5' to 3')	Reverse primer sequence (5' to 3')
SIRT1	ACTCTTCTGTGATTGCTA	TGCTCTCAACATTCCTAT
NRF2	AGTCTCAATGTTGAATCAGTT	TTAGCCAGATGTCATATAAGTC
NOQ1	CCACTCTACTTTGCTCCA	GCTCCTCTTGAACCTCTT
VEGFR2	AAGCATCAGCATAAGAAGA	ACTGTCTATGGTCAAGGT
PABPN1	AGAGCGACATCATGGTAT	CATCAAGGTCATCTTCTGTT
Mt-COX1	AGTAATACCTATAATAATTGGA	AAAGGAGTAGAAATGATG
Mt-ND1	CTTATCCGTCCTCCTAAT	AATGTATCATATTATGGCTATTG
UBC	CTCGTACCTTTCTCACCACAGT	GACACCTCCCCATCAAACCC

Supplementary Figure S1. Serum creatinine (sCr, A) and creatinine clearance (CrCl, B) on day of surgery (Day 56) and 1 day (Day 57), 1 week (Day 63) and 6 weeks (Day 98) after surgery in Sham + vehicle (Veh), ischemia-reperfusion injury (IRI) + Veh, IRI + nicotinamide riboside (NR), IRI+SkQR1 and IRI+NR+SkQR1 animals (n=4-6 for each group). Means and standard deviations are shown (n=4-6 each group). * $p < 0.05$

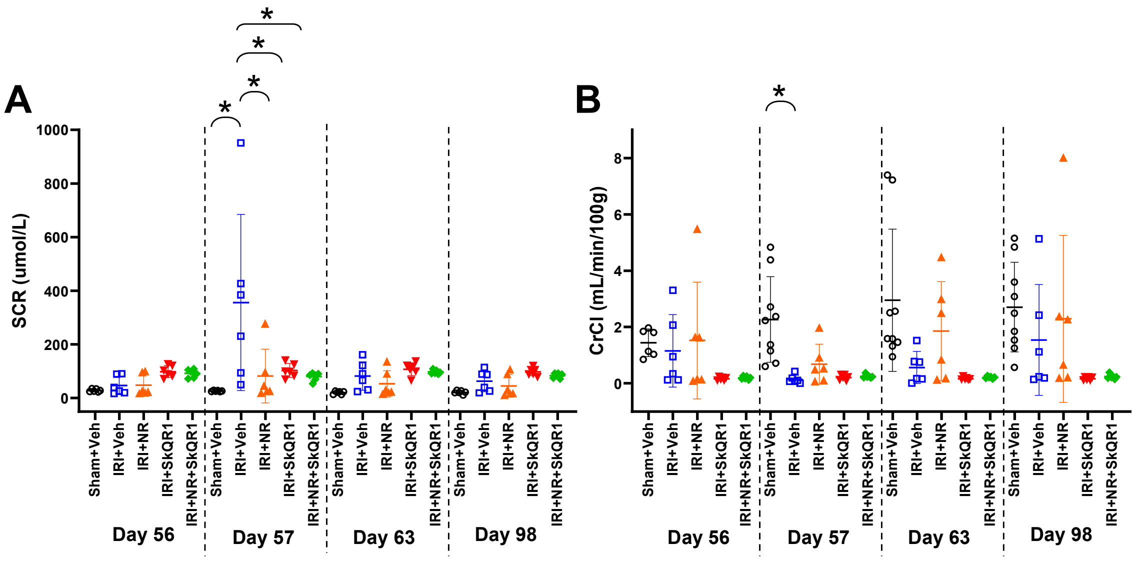

Supplementary Figure S2. Kidney functional reserve (KFR, A), stimulated glomerule filtration rate (GFR, B), unstimulated GFR (C) and relative blood volume (rBV, D) on Day 56 and 98 in Sham + vehicle (Veh), ischemia-reperfusion injury (IRI) + Veh, IRI + nicotinamide riboside (NR), IRI+SkQR1 and IRI+NR+SkQR1 animals (n=6 for each group) shown as means and standard deviations.

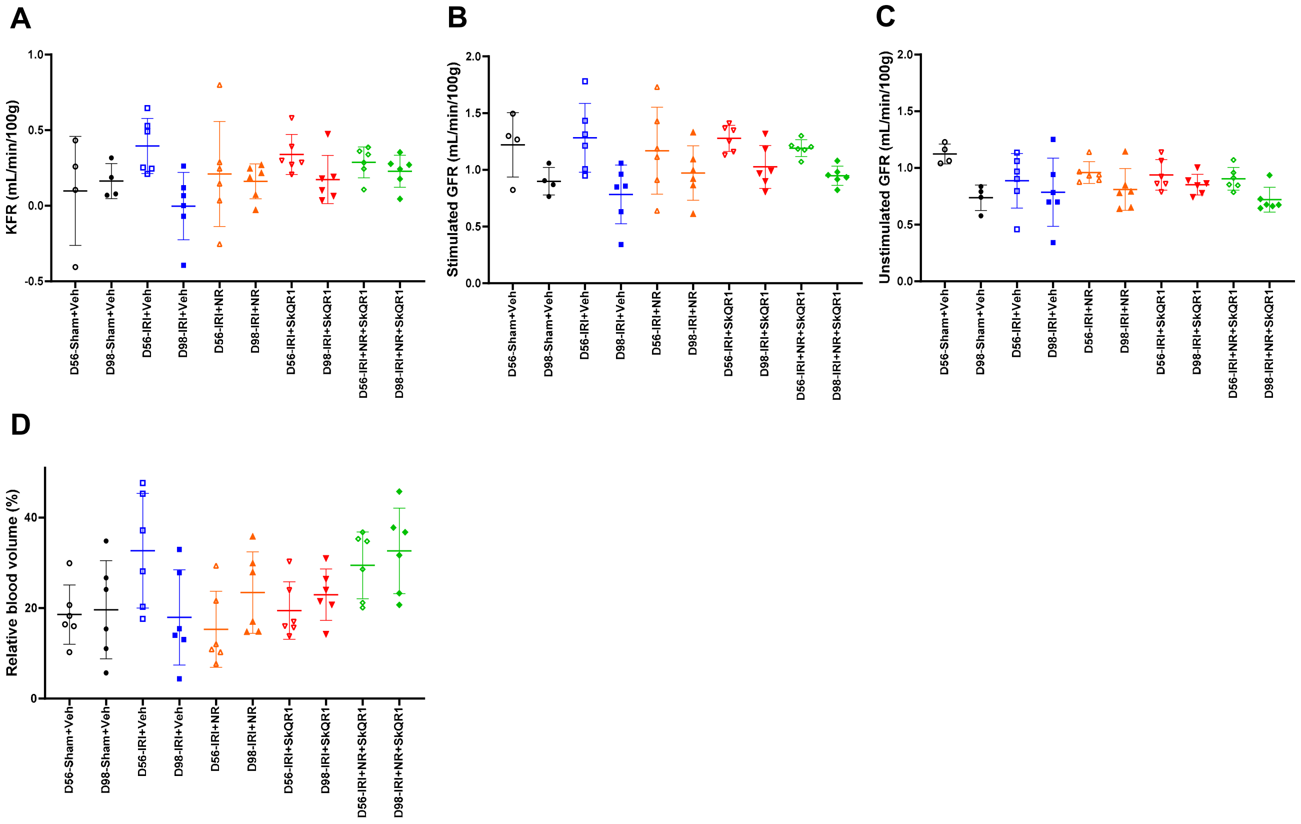

Supplementary Figure S3. Details of experimental protocol. After animals were acclimatised for 1 week, subclinical chronic kidney disease (sCKD) was induced by 0.25% adenine supplemented chow for 28 days followed 28 days of normal chow (n=24). Animals were then randomly assigned to the following procedures and treatments: ischemia-reperfusion injury (IRI) (n=18) or Sham surgery (n=6) on day 56. Nicotinamide riboside (NR) was provided daily from Day 53 to 98. SkQR1 (100 nmol/kg) or an equal volume of SkQR1 vehicle was administered intraperitoneally 3 hours prior to sham or IRI surgery on Day 56 and then 1 hour, 24, 48 and 72 hours post-surgery. Sham-operated animals were administered the SkQR1 vehicle by IP and fed normal chow (Sham+Veh, n=6). IRI-operated animals were either: administered the SkQR1 vehicle and fed normal chow (IRI+Veh, n=6), administered SkQR1 vehicle and fed NR diet (IRI+NR, n=6), administered SkQR1 and fed normal chow (IRI+SkQR1) or administered SkQR1 and fed NR diet (IRI+NR+SkQR1, n=6). This was a unblinded study and all collected data points were used for all analyses, no data points were excluded.

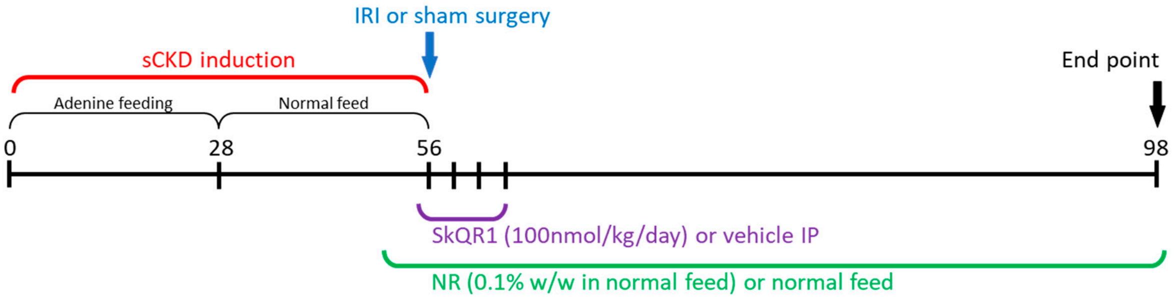

sCr, CrCl, GFR and urine biomarker measurements from IRI+Veh on Day 56, 57 and 63 (n=6), Sham+Veh on Day 56, 57 and 63 (n=6), IRI+NR on Day 56 (n=6), IRI+SkQR1 on Day 56 (n=6) and IRI+SkQR1+NR on Day 56 (n=6) were previously published as part of a larger cohort (n = 51) in Taylor et al (2023). sCr, CrCl, GFR and urine biomarker measurements from Day 57, 63 and 98 of treatment groups (SkQR1, NR and SkQR1+NR) and Day 98 of control groups (Sham+Veh and IRI+Veh) have not been published and all analyses in this study are new findings. All data from histopathology, immunofluorescence, vascular ultrasound, NAD and qPCR assays are new findings.
